# Fluid resuscitation based on dynamic predictors of fluid responsiveness: closed loop algorithm versus anesthesiologists

**DOI:** 10.1186/cc9471

**Published:** 2011-03-11

**Authors:** J Rinehart, B Alexander, L Meng, M Cannesson

**Affiliations:** 1 University of California Irvine, Orange, CA, USA

## Introduction

Closed-loop management of fluid resuscitation has historically been difficult. Given the dynamic predictors of fluid responsiveness, automated management is now feasible. We present simulation data for a novel patient-adaptive closed-loop fluid management algorithm using pulse pressure variation (PPV) as the input variable.

## Methods

Using a simulator that includes physiologic PPV output, 20 practicing anesthesiology residents and faculty were asked to manage fluids and pressors for a 1-hour simulated hemorrhage case of 2 l blood loss over 20 minutes (group 1). One week later, they repeated the simulation, but this time fluids were secretly managed by the closed-loop system while practitioner fluid administrations were ignored and only the pressors were entered (group 2). The simulation was also run 20 times with only the closed-loop (group 3) and 20 times with no management (group 4).

## Results

**Figure 1:**
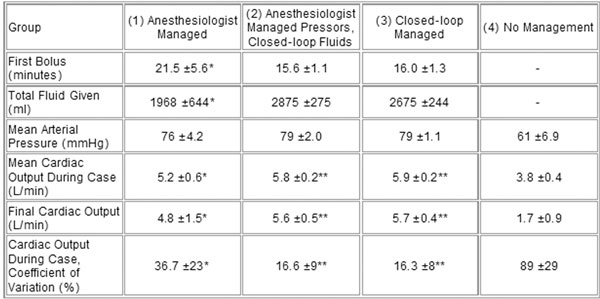
**Data are mean ± SD**. *P < 0.05 versus groups 2, 3, 4; **P < 0.05 versus groups 1 and 4.

Conditions across all groups were similar at baseline for simulated patient weight, height, heart rate (HR), mean arterial pressure (MAP), and cardiac output (CO). Once the hemorrhage began, the closed loop groups (2 and 3) intervened significantly earlier than the practitioners (group 1) and gave more fluid. The mean and final CO was higher in both closed-loop groups than in the practitioner group, and the coefficient of variance was lower. There was no difference in MAP between intervention groups, but all were significantly higher than the unmanaged group. See Figure 1.

## Conclusions

Our data demonstrate that closed-loop management of fluid resuscitation is feasible using our novel dynamic-parameter based algorithm and that this approach can be used to optimize cardiac output.

